# Insights into the Pathogenesis of Viral Haemorrhagic Fever Based on Virus Tropism and Tissue Lesions of Natural Rift Valley Fever

**DOI:** 10.3390/v13040709

**Published:** 2021-04-20

**Authors:** Lieza Odendaal, A Sally Davis, Estelle H Venter

**Affiliations:** 1 Department of Paraclinical Sciences, Faculty of Veterinary Science, University of Pretoria, Onderstepoort, Pretoria 0002, South Africa; 2 Department of Diagnostic Medicine/Pathobiology, College of Veterinary Medicine, Kansas State University, Manhattan, KS 66506, USA; 3 Department of Veterinary Tropical Diseases, Faculty of Veterinary Science, University of Pretoria, Pretoria 0002, South Africa; Estelle.venter@jcu.edu.au; 4 College of Public Health Medical and Veterinary Sciences, Discipline Veterinary Science, James Cook University, Townsville, QLD 4811, Australia

**Keywords:** Rift Valley fever phlebovirus, *Bunyavirales*, emerging diseases, pathogenesis, pathology, tropism, zoonotic disease, arbovirus

## Abstract

Rift Valley fever phlebovirus (RVFV) infects humans and a wide range of ungulates and historically has caused devastating epidemics in Africa and the Arabian Peninsula. Lesions of naturally infected cases of Rift Valley fever (RVF) have only been described in detail in sheep with a few reports concerning cattle and humans. The most frequently observed lesion in both ruminants and humans is randomly distributed necrosis, particularly in the liver. Lesions supportive of vascular endothelial injury are also present and include mild hydropericardium, hydrothorax and ascites; marked pulmonary congestion and oedema; lymph node congestion and oedema; and haemorrhages in many tissues. Although a complete understanding of RVF pathogenesis is still lacking, antigen-presenting cells in the skin are likely the early targets of the virus. Following suppression of type I IFN production and necrosis of dermal cells, RVFV spreads systemically, resulting in infection and necrosis of other cells in a variety of organs. Failure of both the innate and adaptive immune responses to control infection is exacerbated by apoptosis of lymphocytes. An excessive pro-inflammatory cytokine and chemokine response leads to microcirculatory dysfunction. Additionally, impairment of the coagulation system results in widespread haemorrhages. Fatal outcomes result from multiorgan failure, oedema in many organs (including the lungs and brain), hypotension, and circulatory shock. Here, we summarize current understanding of RVF cellular tropism as informed by lesions caused by natural infections. We specifically examine how extant knowledge informs current understanding regarding pathogenesis of the haemorrhagic fever form of RVF, identifying opportunities for future research.

## 1. Introduction

*Rift Valley fever phlebovirus* (RVFV) is a significant veterinary and public health threat that has caused widespread outbreaks of disease in livestock and humans in most countries in Africa and since 2000 in the Arabian Peninsula, specifically Yemen and Saudi Arabia [1]. It is caused by a mosquito-borne RNA virus of the order *Bunyavirales*, family *Phenuiviridae.* genus *Phlebovirus* [2]. During years of abnormally high rainfall, vast swarms of mosquitoes, mainly of the genera *Aedes* and *Culex*, emerge from standing water, and with sufficient numbers of susceptible unvaccinated livestock in the same area as RVFV-infected mosquitoes, epidemics commence [3]. Besides transmission by mosquito bite, exposure to blood and tissues of infected animals can transmit RVFV [4,5,6].

Recurrent epidemics of Rift Valley fever (RVF) have been reported in most countries in eastern and southern Africa since 1931 [7]. Livestock trade contributes to the spread of the disease into disease-free areas thereby expanding the geographical distribution of RVF [8]. Between 1977 and 2007 the disease spread beyond East Africa into Egypt, Mauritania, Somalia, and Sudan [9,10,11,12,13,14,15]. The disease has also spread to Madagascar, Saudi Arabia, Yemen, the Comoros, and Mayotte [16,17,18,19].

Additionally, interepidemic circulation of RVFV is suspected to occur. Data from seroprevalence studies, published for several African countries, suggest that the virus might be maintained at low levels in the environment, with sporadic clinical cases overlooked or misdiagnosed [20]. A review of surveys conducted before 2000, demonstrates significant high-prevalence clusters in areas that have experienced epidemics during the late 20th century and significant low-prevalence clusters in contiguous areas of Western and Central Africa [20]. More recently in South Africa, high seroprevalence was found in cattle (34%) and goats (31.7%) in northern KwaZulu-Natal Province, just south of the Mozambique border, and seroconversions to RVFV were detected throughout the year [21]. Seroprevalence in cattle, sheep, and goats in the Free State Province of South Africa is estimated as 42.9%, 28.0%, and 9.3%, respectively, and the presence of anti-RVFV IgG among domestic ruminants, born after the most recent outbreak, indicates the possibility that virus circulation has occurred during the inter-epidemic period [22]. In northern Kenya, antibodies to RVFV were detected in samples from children born after the outbreak in 1997–1998 indicating that low levels of transmission to humans continued in the interepidemic period [23].

Another notable example is Uganda, which before 2016 had no reports of RVF outbreaks despite its proximity to Kenya and Tanzania where large epidemics in humans and animals had occurred [24]. A study involving 2700 goats in southern Uganda in 2013 reported a seroprevalence of anti-RVFV IgG of 9.8% and the presence of RVF virus neutralizing antibodies (up to 1:80), suggesting that RVF was endemic at least in goats [25]. Subsequently, between 2016 and 2018, ten outbreaks of RVF were reported in Uganda, where cattle, goats, and sheep sampled in 2016 had a seroprevalence of 27%, 7%, and 4% respectively [24]. Another study conducted from 2007 to 2008 on the shores of Lake Malawi in south-western Tanzania demonstrated a seroprevalence of 29.3% in 17,000 human subjects tested, with much lower rates in areas distant from the lake [26]. A recent study of 2014 and 2015 samples from the Democratic Republic of Congo reveals a country-wide distribution of RVF with seroprevalence in cattle ranging from 16.16% in the mountainous zones to 7.34% in the forest zones [27]. Although Rift Valley fever disease or outbreaks have not been observed in south-western Tanzania or the Democratic Republic of Congo, these findings suggest that the virus is circulating, and that the occasional occurrence of disease is missed [27]. Therefore, sporadic clinical cases, not linked with high rainfall, in both animals and humans may be overlooked or misdiagnosed in many countries in Africa as well as endemic areas of eastern and southern Africa.

Rift Valley fever (RVF) mainly affects sheep but cattle, goats, camelids, and other wild ruminants, particularly African buffaloes, are also susceptible [28]. The onset of disease in cattle and sheep is marked by the onset of pyrexia, and may be accompanied by anorexia, weakness, listlessness, a nasal discharge, diarrhoea and occasionally haematochezia and haematuria [29]. Peracute disease occurs in lambs and calves less than two-weeks-old, with estimated mortality as high as 90% to 100% in lambs and 10% to 70% in calves [30]. Mature livestock are significantly less susceptible to fatal disease with mortalities of approximately 10% to 30% in sheep and 5% to 10% in cattle [30]. In camels, RVF can cause abortions and neonatal deaths, whereas infected wild ruminants are usually clinically asymptomatic [30,31].

Epidemics are also characterised by near-simultaneous abortions of pregnant domestic ruminants, African buffaloes, and camels [29,32,33]. Whereas reports regarding abortions in cattle, camels, and African buffaloes infected with RVFV are scant, multiple studies have demonstrated a wide variety of outcomes for pregnant ewes and their foetuses [32,34,35,36,37]. Large numbers of pregnant ewes abort (90–100%) [30]. Ewes in the later stages of pregnancy may be more susceptible to lethal disease and die before aborting, whereas ewes in the earlier stages of pregnancy may abort or resorb the foetus and survive the infection [32,34,38,39]. Occasionally, RVFV infected ewes show no clinical signs, do not seroconvert, do not have a detectable viraemia and lack lesions typical of RVF [34]. Similarly, in approximately 20% of ovine foetuses, the rapid progression of placental necrosis causes foetal mortality before foetal organs become infected [37,40]. Therefore, it may not be possible to rule out RVF in individual cases even if sampling is adequate.

Most human infections with RVFV present as an uncomplicated acute febrile illness. However, in a minority of patients, severe hepatic disease with haemorrhagic manifestations, renal impairment, encephalitis, and ocular lesions can complicate illness [41]. Additionally, RVFV can replicate in the syncytiotrophoblast layer of the human placenta and its vertical transmission correlates with an increased risk of miscarriage in humans [41,42,43,44].

Available literature about the viral haemorrhagic form of RVF is scant. Here, through an examination of RVFV’s cellular tropism, and its implications specifically for this manifestation of RVF, we identify knowledge gaps and several warranted avenues for future research. First, we present an overview of the virus. We follow this with a discussion of RVFV’s cellular tropism as demonstrated in examinations of macro- and microscopic lesions in animals and humans who succumbed to natural RVFV infections. Accompanying this section are a series of images derived from cases we published prior [37,45,46]. Information regarding these—including immunohistochemistry methods—are already available [47]. We use standard special stains to highlight additional features relevant to this discussion. Finally, we examine RVF within the context of what is known about other viral haemorrhagic fevers including dengue haemorrhagic fever, yellow fever, Crimean-Congo haemorrhagic fever, Marburg, Ebola and Lassa, since there are many similarities between the pathology and pathogenesis of these diseases and RVF.

## 2. Aetiological Agent

### 2.1. Structure and Replication

Rift Valley fever virus is an enveloped, single-stranded RNA virus with only one serotype and multiple lineages [48,49,50]. The viral genome consists of three segments, large, medium, and small (L, M, and S) [51]. Similar to other arboviruses such as bluetongue and epizootic haemorrhagic disease virus, RVFV’s genome has low substitution rates, with genomic diversity largely driven by reassortment [49]. The L RNA segment codes for the viral RNA-dependent RNA polymerase while the M segment codes for the two envelope glycoproteins, carboxy-terminus glycoprotein (GC) and amino terminus glycoprotein (GN), as well as two non-structural proteins, NSm1 and NSm2 [52]. The S segment codes for the viral nucleocapsid protein (N) and the nucleus-associated non-structural (NSs) protein [51,53].

Following replication in the cytoplasm, the three viral RNA segments form ribonucleoprotein complexes with the viral N protein and RNA polymerase [51,54]. The N protein encapsidates the viral genome forming an N-RNA multimer and is also required for its replication and transcription by the RNA polymerase [55]. Late in the infection, GC combines with GN, forming a polyprotein that localizes to the Golgi complex where the ribonucleoprotein complexes are also recruited by the glycoproteins [51,54]. The Golgi complex undergoes morphological changes with vacuolization and dispersion of small and large virus component laden vesicles in the cytoplasm, which fuse with the plasma membrane allowing for the release of the virus into the extracellular environment [56]. The released spherical, enveloped virions measure 90–110 nm in diameter and are further protected by a proposed T = 12 icosahedral glycoprotein layer [57,58,59]. The cycle repeats after virus entry into a variety of cells, a process which is mediated by the glycoproteins binding to cellular receptors, followed by uptake facilitated by ribonuclease kappa (RNASEK) [1,60].

### 2.2. Cellular Receptors

In vitro experiments with human monocyte-derived dendritic cells demonstrate binding and internalization of RVFV by Dendritic Cell-Specific Intercellular adhesion molecule-3-Grabbing Non-integrin (DC-SIGN) [61]. RVFV glycoproteins also interact with DC-SIGN, a multifunctional C-type lectin receptor on antigen-presenting cells that interacts with Intercellular Adhesion Molecule-3 (ICAM-3) on T lymphocytes during their activation [62]. Therefore, DC-SIGN expressed on the surface of dermal dendritic cells possibly plays a critical role in the initial transmission of RVFV following an insect bite or mucosa exposure [61]. However, this remains to be confirmed in vivo.

Also, RVFV infects other cell types, most of which do not express DC-SIGN (e.g., hepatocytes, adrenocortical cells, renal tubular epithelial cells). Heparan sulfate (HS) has also been shown to facilitate RVFV entry into cells [63]. However, in the absence of HS, Chinese hamster ovary cells are still permissible to RVFV, albeit at lower levels [63]. Hence, another yet to be identified cellular receptor must be involved [51,61]. Liver/Lymph-Specific Intercellular adhesion molecule-3-Grabbing Non-integrin (L-SIGN), a homologous molecule to DC-SIGN with similar functional interactions with ICAM-3, might play a role in the liver tropism of RVFV [64,65,66]. However, L-SIGN is expressed on liver sinusoidal endothelial cells, not hepatocytes, and acts as an attachment/capture receptor rather than an endocytic receptor. Therefore, the details of RVFV entry into hepatocytes remain to be clarified.

Recent studies demonstrated that the cellular tropism of RVFV coincides with the distribution of class AI scavenger receptors (SCARA1) in tissues [45,67]. Immunolabelling for RVFV was detected in vascular smooth muscle cells, endothelial cells and tissue macrophages in a variety of tissues (liver, spleen, lungs, kidneys, lymph nodes, intestinal tract, skin, and testis) all of which are known to express SCARA1 [45,68]. Uptake of adenovirus 5, herpes simplex virus type 1 and vaccinia virus can be mediated via class A scavenger receptors [69,70,71]. These findings suggest that following the initial transmission of RVFV by dendritic cells in the skin, SCARA1 might play a secondary role in further rounds of RVFV infection in other cells and tissues.

### 2.3. Role of the Non-Structural Proteins

The virus’ NSs protein has many biological functions in infected cells, which include modulating the interferon response, facilitating efficient viral translation, and acting as a general inhibitor of transcription [72]. The host’s antiviral interferon (IFN) system is counteracted by the NSs protein, which prevents nuclear activation of the IFN-β gene and the cytoplasmic transcription of interferon mRNAs [73,74]. As a result, unrestricted viral replication is promoted. Additionally, nuclear NSs filaments bind to Sin3A Associated Protein 30 (SAP30) and to Yin Yang 1 (the activator/repressor of interferon transcription), forming a multiprotein repression complex on the IFN-β promoter, thereby blocking interferon expression [75].

The NSs protein also allows for efficient viral translation and replication in infected cells by downregulating the activation of protein kinase R (PKR) [73]. Protein kinase R is activated by dsRNA, introduced to cells during viral replication, and once active, PKR phosphorylates eukaryotic initiation factor 2 alpha (eIF2α), thereby suppressing viral translation. In RVFV-infected cells, the NSs protein decreases PKR abundance and prevents eIF2α phosphorylation, which augments viral replication [76].

The NSs protein also inhibits general transcription activity in cells by directly interacting with components of the basal transcription factor II human (TFIIH) [74,77]. The p44 subunit of the TFIIH complex is sequestered by the NSs filamentous structure, thereby blocking assembly of TFIIH and drastically reducing transcriptional activity in RVFV-infected cells [74]. In addition, NSs protein interacts with the TFIIH subunit p62 and promotes its post-translational degradation [77]. The presence of NSs filaments in the nucleus of infected cells also induces DNA damage responses and causes cell-cycle arrest which causes the activation of p53 and apoptosis [78].

The NSs also interacts with the DNA damage response system (DDR) [78]. Specifically, it induces up-regulation of the ataxia-telangiectasia mutated kinase (ATM) arm of the DDR, which responds to double-stranded DNA breaks, by causing cell-cycle arrest [78]. In contrast, it down-regulates the Rad3-related kinase (ATR) pathway of the DDR, which senses single-stranded DNA breaks and likely has an antiviral role [78]. Through this double interaction with the DDR, NSs promotes viral replication.

Induction of apoptosis in virus-infected cells, and subsequent phagocytosis of these cells, is one of the host defence mechanisms that eliminates infected cells thereby limiting viral replication and spread [79]. It has been demonstrated that RVFV triggers apoptosis mainly through activation of caspase-8 [80]. The NSm protein of RVFV suppresses apoptosis in target cells [80]. It suppresses the expression of caspase-8 in infected cells thereby delaying apoptosis and ensuring the efficient release of progeny RVFV within the first 24 h after infection [80]. As a result, most of the progeny virus is released before virus-induced apoptosis occurs. Without the NSm protein, RVFV replicates poorly in murine macrophages and has attenuated virulence in mice compared to the parental virus, indicating NSm has a role in viral replication in mammals [81].

Virus-infected cells that undergo transcriptional or translational suppression due to non-structural protein activity may also be exposed to tumour necrosis factor-alpha (TNF-α) and rapidly die by apoptosis [80]. Most humans and animals do not develop fatal RVF, presumably due to variations in the host’s innate immune responses and exposure of the infected cells to extracellular factors, such as TNF-α, that induce apoptosis before the completion of maximum progeny virus production [80]. This process may prevent severe, nearly diffuse necrosis of cells in some species or age groups, preventing the death of the host and promoting the completion of the life cycle of the virus [80].

## 3. Macro- and Microscopic Lesions and Virus Tropism in Natural Infections

### 3.1. Overview

Although RVF is known to occur in humans, a wide range of domestic and wild ruminants, and camelids, lesions of naturally infected cases of RVF have only been described in detail in sheep with a few reports concerning cattle and humans [32,35,37,45,46,47,82,83,84,85,86,87,88]. While lesions can be seen throughout the body, multifocal, randomly distributed necrosis is the most commonly observed lesion in both ruminants and humans. Descriptions of the lesions in natural infections of RVF are similar in humans and ruminants except for encephalitis and retinitis that occurs in humans but has not been described in ruminants. Furthermore, detailed studies of statistically relevant numbers of sheep and observations in other ruminants reveal that lesions in neonatal ruminants (<1-month-old) and foetuses differ from those observed in adult ruminants, with the most prominent differences noted in the liver, kidneys, and lymphoid tissues [1,37,45,46,89]. While there is no clear explanation for these differences, they may be due to age-related susceptibility of the primary target cells of RVFV [1,37,45,46,89]. Immunohistochemistry based examination of the tissue and cellular tropism of RVFV in naturally infected sheep reveals viral antigen-positive hepatocytes, renal tubular epithelial cells, renal juxtaglomerular and extraglomerular mesangial cells, adrenocortical epithelial cells, cardiomyocytes, Purkinje fibres, skeletal muscle cells, epidermal keratinocytes, endothelial cells, vascular smooth muscle cells, tissue macrophages, and neutrophils and further highlights the aforementioned age-related differences discussed more exhaustively by organ type below [37,45,46].

In adult ruminants and neonates, lesions suggestive of vascular endothelial injury include mild hydropericardium, hydrothorax and ascites, marked pulmonary congestion and oedema, congestion and oedema of lymph nodes, and haemorrhages in many tissues [45,46]. Conversely, in foetuses, haemorrhages are minimal but plasma leakage with effusions in body cavities, accompanied by severe brain and lung oedema, is common [37]. Occasionally, the rapid progression of placental necrosis causes foetal mortality before other foetal tissues can be infected [40]. Notably, foetal malformations have not been reported in natural cases of RVF or in experimental cases using wild-type virus [32,34,37,38,40]. Instead, ill-advised use of live attenuated vaccines in ruminants in the first or second trimester of pregnancy should be investigated if foetal malformations are reported [90,91,92,93].

### 3.2. Liver

**Figure 1:**
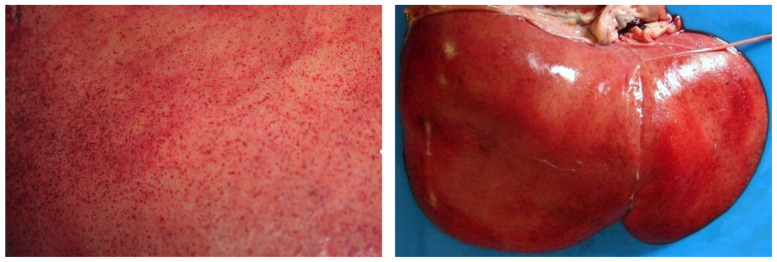
Macroscopic pathology of the liver in sheep naturally infected with RVFV. (**A**) Innumerable petechia on the parietal surface of the liver of an adult sheep. (**B**) Liver of a new-born lamb with extensive necrosis and pinpoint subcapsular petechia giving the liver a pale yellow to red mottled appearance.

**Figure 2:**
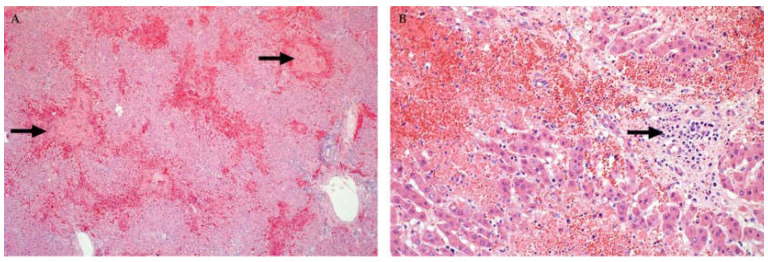
Histopathology of the liver of cattle naturally infected with RVFV (Haematoxylin and eosin (H&E) stain). (**A**) Random multifocal to coalescing necrosis and haemorrhage (arrows), original magnification (mag) 40×. (**B**) Necrosis of hepatocytes extends into the periportal zones, specifically affecting hepatocytes of the limiting plate. Mild mononuclear cell inflammation (arrow) is also present in the portal area, mag 200×.

In adult cattle and sheep, innumerable petechia may be present on the parietal and cut surfaces of the liver (Figure 1) and occasionally, there are mural haemorrhages and intraluminal blood in the gall bladder [32,35,45,86]. Histologically, foci of necrosis are situated irregularly throughout the lobule in adult ruminants and might involve up to two-thirds or more of the lobule (Figure 2). A diagnostically significant finding is that necrosis often extends into the periportal zones, affecting hepatocytes of the limiting plate which is typically not seen with liver injury due to hepatotoxins [45]. Haemorrhage and a mild to moderate infiltrate of neutrophils, lymphocytes, and macrophages are generally also present in and surrounding necrotic areas [45]. This is accompanied by a mild infiltrate of mononuclear inflammatory cells in the portal tracts, with occasional karyorrhexis of mononuclear cells in the portal interstitium [45].

In calves and lambs less than a month-old, pale pinpoint subcapsular petechiae and foci of necrosis may be present in the liver and virtually all hepatocytes undergo necrosis (Figure 1) [32,35,46]. In foetuses, the liver usually does not have any discernible macroscopic lesions [37]. However, microscopically there is random dropout of hepatocytes from the reticulin framework which ranges from minimal to nearly diffuse hepatocyte necrosis. Haemorrhage or pooling of blood in spaces depleted of hepatocytes is inconspicuous in foetuses whereas oedema is present in the portal areas in most cases (Figure 2) [37]. Bile stasis is not present in any age category and bile ducts are not involved [32,35,37,45,46].

**Figure 3:**
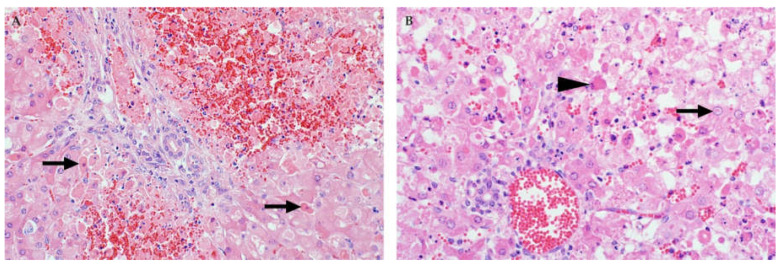
Histopathology of the liver in young lambs naturally infected with RVFV (H&E stain). (**A**) Hepatocytes with features of apoptosis (arrows), characterized by disassociation of cells, hypereosinophilic cytoplasm and pyknosis or karyorrhexis, mag 400×. (**B**) Diffuse necrosis of hepatocytes, filamentous eosinophilic intranuclear inclusions (arrow), and late apoptotic bodies (arrowhead), magnification 600×.

Injured hepatocytes in all age groups often have features of apoptosis (Figure 3) [32,35,37,45,46,82]. Features of early apoptotic cells (also referred to as Councilman bodies or acidophilic bodies) include dissociation of cells, cellular shrinkage and rounding, hypereosinophilic cytoplasm, pyknosis (nuclear chromatin condensation) and karyorrhexis (nuclear fragmentation) [45]. Early apoptotic bodies fragment into multiple smaller (late) apoptotic bodies that are eosinophilic fragments of cytoplasm of varying sizes, which may or may not contain nuclear fragments [45]. However, a lytic cell death mechanism (i.e., necroptosis or pyroptosis) may also be involved in the pathogenesis of cell death and this is characterized by cell swelling, rupture of the plasma membrane, and cellular collapse [46]. The latter is especially conspicuous in neonates where many hepatocytes are severely swollen and rounded with complete disintegration of the nucleus (karyolysis) or only small nuclear fragments remaining (karyorrhexis) [46].

Amongst the *Phenuiviridae*, RVFV is unique in that the NSs protein forms ribbon-like filaments in the nucleus even though the virus replicates in the cytoplasm of host cells [53]. In haematoxylin-and-eosin-stained tissue sections these rod-shaped and eosinophilic intranuclear inclusions are a significant diagnosis-specific indicator [35,37,45,46]. A recent study showed that intranuclear inclusions were detectable in 62% of ovine foetal liver specimens and 38% of neonatal lamb livers [37,46]. In contrast, these inclusions are of limited diagnostic value in adult ruminants and calves where they are often not identifiable and may be confused with pseudo-inclusions.

**Figure 4:**
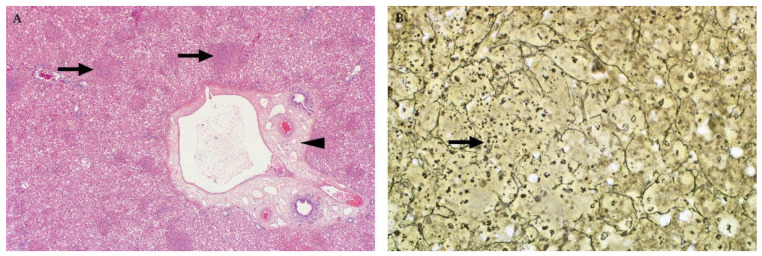
Histopathology of the liver in sheep foetuses naturally infected with RVFV. (**A**) Multiple foci of liquefactive hepatic necrosis (arrows), also referred to as primary foci, are present (H&E stain). There is also severe periportal oedema (arrowhead), mag 40×. (**B**) Nuclear fragments and remnants of reticulin fibres (arrow) are present in an area of liquefactive necrosis (Gordon and Sweets’ silver stain for reticular fibres with Van Gieson counterstain), mag 400×.

A very distinctive feature in ruminant neonates and foetuses is discrete randomly distributed foci of liquefactive hepatic necrosis (also referred to as primary foci) against a background of diffuse hepatocellular death (Figure 4) [35,37,46,82,83]. Primary foci vary in size and number and consist of lysed hepatocytes and infiltrating neutrophils and macrophages that undergo degenerative changes and likewise disintegrate. This results in myriads of small nuclear and cytoplasmic remnants within a collapsed reticulum framework (Figure 4). It is important to emphasize, however, that the absence of necrosis, primary foci, or intranuclear inclusions in the liver of foetuses does not necessarily exclude a diagnosis of RVF [37].

The hepatic lesions in humans are similar to those in ruminants. In humans, foci of hepatic necrosis are associated with haemorrhage and involve the mid to central zones of the hepatic lobule, and often extend peripherally to the portal tracts [88]. Hepatic necrosis may also be diffuse [84]. Councilman bodies (early apoptotic bodies) and a mild inflammatory infiltrate of predominantly lymphocytes and macrophages with a few neutrophils, and a lack of bile stasis are also described in RVF hepatic pathology in humans [84,88].

**Figure 5:**
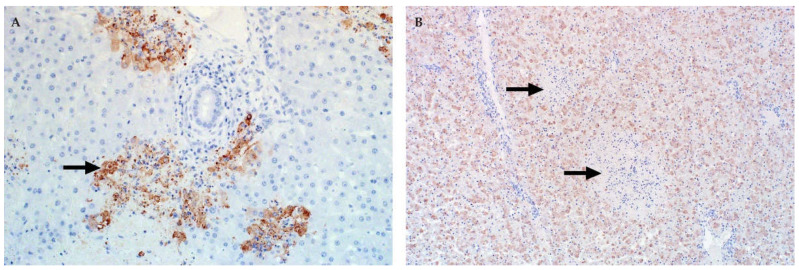
Immunolabelling for RVFV in the liver of sheep (polyclonal mouse anti-RVFV nucleoprotein antibody, avidin-biotin-peroxidase detection system, NovaRED chromogen and haematoxylin counterstain). (**A**) In this specimen from an adult sheep, viral antigen (arrow) is present in multiple foci in the lobule. Labelling is present in the cytoplasm of injured hepatocytes and is diffuse or fine granular, mag 400×. (**B**) In young lambs, viral antigen is diffusely present in the cytoplasm of hepatocytes, but labelling is sparse in primary foci (arrows), mag 100×.

In sheep, an anti-RVFV nucleoprotein antibody (Figure 5) reveals viral antigen in the cytoplasm of injured hepatocytes, and in cytoplasmic fragments within the sinusoids and central veins [37,45,46]. Scattered viral antigen-positive Kupffer cells and neutrophils are also present.

### 3.3. Kidney

**Figure 6:**
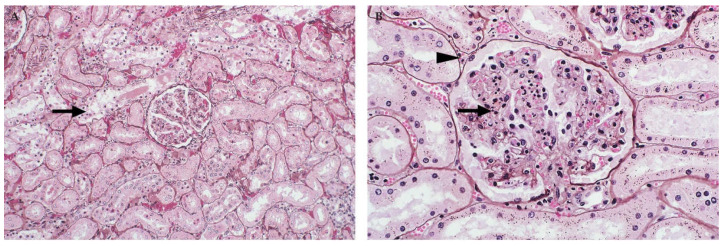
Kidney of RVFV-infected adult sheep (Jones’ methenamine silver stain). (**A**) Lesions in necrotic tubules are characterized by tubular epithelial cell pyknosis, karyorrhexis, and karyolysis accompanied by detachment of the epithelium from the basement membrane (arrow), mag 200×. (**B**) Pyknosis and karyorrhexis are present in a renal glomerulus (arrow) with a marked decrease in mesangial cellularity. Pyknosis is also present in the interstitium (arrowhead) and in many tubular epithelial cells, mag 400×.

Macroscopically, in the kidneys a few small cortical haemorrhages may be present [32,45,82]. Microscopically, adult sheep, cattle, and calves frequently have a severe nephrosis (Figure 6) [32,35,45,94,95]. Conversely, in young lambs (< 1-month-old) and foetuses, the kidney lesion rarely progresses beyond tubular epithelial cell degeneration [32,37,46,82].

Irrespective of age, the glomeruli also appear less densely cellular than normal [37,45,46]. Additionally, scattered pyknosis and karyorrhexis is present within the glomeruli of most cases (Figure 6) and nuclear debris is also often present in the interstitial capillaries [37,45,46,82,83].

Similarly, in human patients that died from RVF, subcapsular renal haemorrhages, degeneration of proximal tubular epithelial cells and a slight infiltrate of cells in the glomeruli are described [88]. Moreover, focal renal tubular epithelial necrosis with interstitial inflammation and intratubular casts were reported for a single human kidney sample [87].

**Figure 7:**
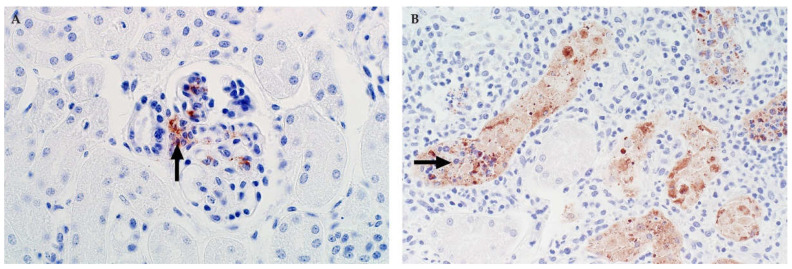
Immunolabelling for RVFV in the kidney of sheep (NovaRed IHC as detailed in Figure 5). (**A**) In this specimen from a young lamb, viral antigen is prominent in Lacis cells in the glomerulus (arrow). Other cells, morphologically consistent with endothelial cells also labelled in the glomerulus. Cells in the macula densa are not labelled, mag 400×. (**B**) Adult sheep with immunolabelling of necrotic renal tubular epithelial cells (arrow) in the renal cortex, mag 600×.

In adult sheep, immunolabeling for RVF viral antigen is most often present in the renal cortex within glomerular and interstitial capillaries, tubular epithelial cells, and vascular smooth muscle cells (Figure 7) [45]. In ovine neonates and foetuses, immunolabelling within blood vessels and capillaries is more extensive, with viral antigen also often present in the medullary interstitial capillaries [37,46]. Immunolabelling is also present at the vascular pole of the glomerulus opposite the macula densa, within a small group of cells that are likely juxtaglomerular and extraglomerular mesangial cells also referred to as Lacis cells (Figure 7). Whereas this is rarely seen in adult sheep it is very common in younger animals. Cells of the macula densa are not affected. Labelling is also more often present in smooth muscle cells in the efferent or afferent arterioles or vascular endothelial cells in neonates and foetuses than in adult animals. RVFV antigen was also detected in a fatal human case in renal tubular epithelial cells [87].

### 3.4. Adrenal Gland

In the adrenal glands, necrosis varies from individual cells to aggregates and is found predominantly in the zona fasciculata, although occasionally cells in the zonae glomerulosa and reticularis are also involved [35,37,45,46,82]. In sheep, irrespective of age, immunolabelling may be present in areas of necrosis or in single cells or small groups of apparently viable cells [45]. In ovine neonates and foetuses, viral antigen may also be present in capillaries in the periadrenal adipose tissues or in blood vessels in the capsule [37,46]. Lesions and viral antigen are absent from the adrenal medulla in all age categories in sheep.

### 3.5. Lymphatic Organs

**Figure 8:**
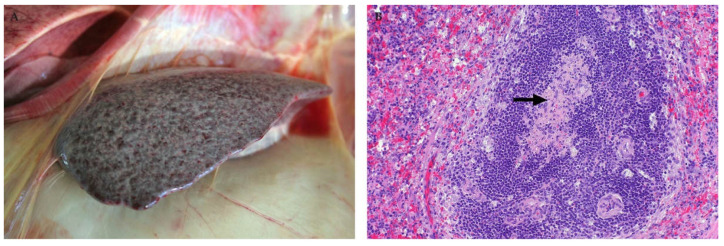
Spleen of an RVFV-infected adult sheep. (**A**) Scattered sub-capsular petechiae are present. (**B**) Lymphocytolysis is most apparent in the germinal centre (arrow) (H&E stain), mag 200×.

Typically, RVFV-infected spleens are not enlarged and occasionally they have sub-capsular petechiae (Figure 8) [32,45,85]. Occasionally, the spleen is also congested [82,83]. Microscopically, there are varying degrees of lymphocytolysis in the white and red pulp, generally giving specimens a paucicellular appearance [35,37,45,46,82,94,95]. In adult ruminants, necrosis is most apparent in the germinal centres, mantle zones, marginal zones, and peripheral zones of the periarteriolar lymphoid sheaths of the white pulp (Figure 8). In young ruminants and foetuses, splenic necrosis is also a prominent feature with lymphocytolysis mainly involving lymphocytes in the red pulp and the peripheral aspects of the periarteriolar lymphoid sheath [46,96]. This is mainly due to the developmentally normal absence or poor development of follicular germinal centres, mantle cell layers, and marginal zones in young ruminants and foetuses. Similar lesions have been reported in humans fatally infected with RVFV, including karyorrhexis and karyolysis of lymphocyte nuclei in the spleen and lymph nodes as well as atrophy of the white pulp [84,88].

**Figure 9:**
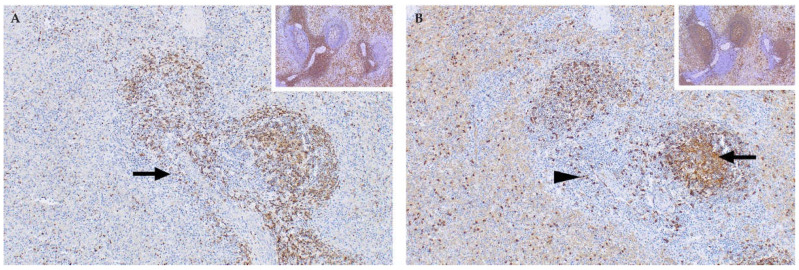
Spleen of an RVFV-infected adult sheep with sequential sections immunolabelled for T and B lymphocytes respectively (polyclonal rabbit anti-CD3 and anti-CD20 antibodies, micro-polymer detection system, DAB chromogen and haematoxylin counterstain). (**A**) Labelling with the anti-CD3 antibody shows a marked loss of T lymphocytes. Only scattered T lymphocytes remain in the red pulp and there is severe depletion of the periarteriolar lymph sheath (arrow), mag 100×. Inset: Spleen from a healthy control sheep showing a normal distribution of abundant T lymphocytes, mag 100×. (**B**) Labelling with the anti-CD 20 antibody shows necrotic debris in the germinal centre (arrow) with a few residual B lymphocytes in the marginal zones (arrowhead) of the periarteriolar lymph sheath and in the red pulp, mag 100×. Inset: Spleen from a healthy sheep with multiple lymphoid follicles that contain many B lymphocytes, mag 100×.

Anti-CD3 and anti-CD20 immunolabelling demonstrate a marked difference in the morphology of the spleen of healthy adult sheep when compared to RVFV infected sheep (Figure 9). The number of B and T lymphocytes are significantly reduced in both the red and white pulp in RVF cases [45]. Germinal centres which contain mostly B lymphocytes, are often collapsed. There is also an abnormal distribution of T lymphocytes, with relatively more than normally expected present in the collapsed germinal centres. However, there is a paucity of T lymphocytes overall. Notably, the antibodies cannot differentiate between viable lymphocytes and dead or dying lymphocytes. Therefore, cellular debris in necrotic germinal centres is often anti-CD20 positive.

Macroscopically, lymph nodes are often enlarged and oedematous with scattered haemorrhages in the cortex and medulla [32,35,82,85]. Enlargement of the mesenteric lymph nodes is frequently emphasised in these reports. In the intestinal tract, lymphoid depletion in the lamina propria is most severe in the distal jejunum and ileum and includes moderate oedema, pyknosis, and karyorrhexis of mononuclear cells in the submucosa, and a mild neutrophilic infiltrate [45,82]. Varying degrees of lymphocyte pyknosis and karyorrhexis are also present in the Peyer’s patches and correspond in degree of intensity to the changes in other lymphoid tissues throughout the body [46,82].

In the thymus, petechiae or ecchymoses are macroscopically visible in neonatal lambs. Histologically, haemorrhages are present in the interstitium. In both young lambs and sheep foetuses, lymphocytolysis, which is present in other lymphoid organs, is absent in the thymus [37,46].

**Figure 10:**
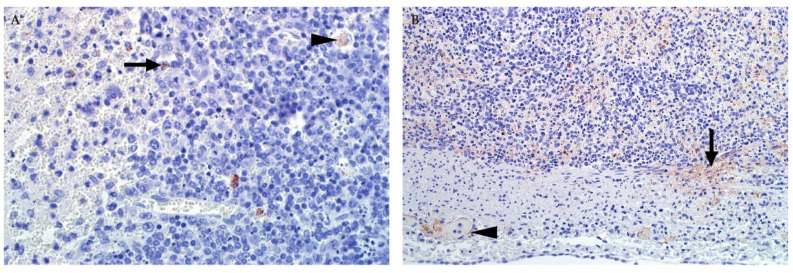
Spleen of a RVFV-infected sheep (NovaRed IHC as detailed in Figure 5). (**A**) In this specimen from an adult sheep, viral antigen is present in multiple macrophages in the marginal zone of the white pulp (arrow). Labelling is also present in a tingible-body macrophage in the white pulp (arrowhead), mag 400×. (**B**) Prominent immunolabelling of non-cell-associated antigen and cellular debris in the subcapsular red pulp (arrow). Viral antigen is also present in endothelial cells (arrowhead) and the capsular smooth myocytes, mag 200×.

In the spleen and lymph nodes, immunolabelling for RVFV is mainly in cellular debris or macrophages, including tingible body macrophages (Figure 10) [45]. In adult sheep, positive labelling for viral antigen is observed more readily in the white pulp, and particularly in the marginal zone of the spleen or the sinusoids of the lymph nodes [45]. In young lambs and foetuses, labelling is also prominent in the subcapsular red pulp and smooth muscle cells within the capsule of the spleen as well as small blood vessels (Figure 10) [46]. Non-cell-associated antigen and antigen in endothelial cells and cellular debris is also often present in small blood vessels in the lymphoid organs, including the thymus, of young lambs and foetuses [37,46]. Labelling is conspicuously absent from thymocytes [37,46].

No convincing evidence of vascular fibrinoid necrosis or microvascular fibrin thrombi is present in the spleen or in any organ in sheep or humans [45,84,88]. However, fibrin deposits have been reported to occur in the red pulp of the spleen of naturally infected cattle and calves and have also been detected in experimentally infected cattle [35,97]. Thrombi or fibrin deposits are also occasionally present in the liver sinusoids or central veins of cattle and within primary foci in calves [35].

### 3.6. Lung

**Figure 11:**
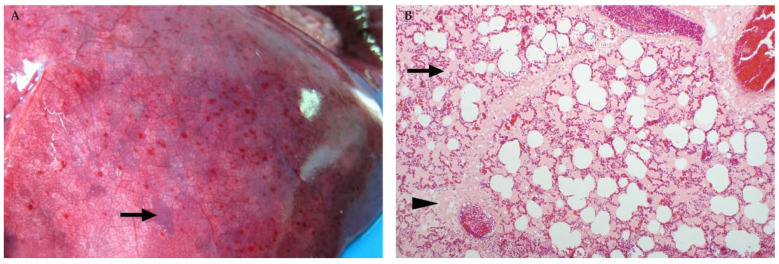
Lungs of RVFV-infected lamb and adult sheep. (**A**) The interstitium in the lungs of this lamb is markedly expanded due to oedema, and dark red areas of atelectasis are present (arrow). Multifocal petechiae are also present in the serosa. (**B**) Microscopically, intra-alveolar (arrow) and interstitial (arrowhead) lung oedema is present in this adult sheep (H&E stain), mag 100×.

In ruminants, marked lung oedema and congestion is a consistent macroscopic finding irrespective of age (Figure 11) [35,37,45,46,82]. The lungs are often wet and heavy, with fluid oozing from the cut surfaces and copious amounts of foam filling the trachea and bronchi. Multifocal haemorrhages, or blood in the trachea and bronchi, may also be present in sheep and cattle [45,82,86]. Microscopically, intra-alveolar and interstitial oedema with atelectasis, emphysema, and occasional haemorrhages are present in adult ruminants and neonates. In foetuses, oedema expands the connective tissue surrounding blood vessels, bronchi, or bronchioles and is also present in the pulmonary septa [37]. Inflammatory cells, varying from low to marked numbers, are present in the alveolar capillaries in all cases, and consist of predominantly mononuclear cells accompanied by fewer neutrophils. Diagnostically significant single cell pyknosis and karyorrhexis are also occasionally present in the alveolar septa, pulmonary blood vessels, and peribronchial lymphoid tissues in all age groups of sheep and cattle [35,45,46,82]. In fatal human RVF cases, there is frank haemorrhage in the lungs, with microscopic alveolar oedema and haemorrhage [88].

In the lungs of sheep, viral antigen is present in pulmonary intravascular macrophages or in the capillaries associated with cellular debris [37,45,46]. Immunolabelling is also present in endothelial cells and vascular smooth muscle cells, but this is more prominent in young lambs and sheep foetuses [37,46].

### 3.7. Heart

Epi- and endo-cardial haemorrhages are present in most cases in ruminants and humans [35,45,46,82,88]. However, histomorphological lesions attributable to RVFV infection are not present in the cardiac parenchyma [35,37,45,46,88].

**Figure 12:**
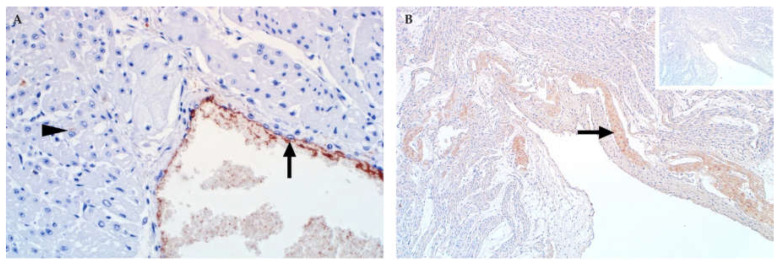
Heart of a RVFV-infected sheep (NovaRed IHC as detailed in Figure 5). (**A**) In this specimen from a young lamb, viral antigen is present in endothelial cells (arrow) and cardiomyocytes (arrowhead), mag 400×. (**B**) Immunolabelling is especially prominent in the Purkinje fibres (arrow), mag 100×. Inset: Negative control using a following slide from the same case (avidin-biotin-peroxidase system using mouse polyclonal anti-Wesselsbron antibody, NovaRED peroxidase substrate with haematoxylin counterstain), mag 100×.

Immunolabelling for RVFV is rare in adult sheep and typically associated with endothelial cells or vascular smooth muscle cells [45]. In neonates—and especially foetuses—labelling in the heart is widespread and involves cardiomyocytes, Purkinje fibres, endothelial cells or cellular debris in small blood vessels and capillaries, as well as in vascular smooth muscle cells of small blood vessels (Figure 12) [37,46]. Diffuse labelling of the myocardium and intense subepi- and endocardial labelling is occasionally observed in foetuses [37].

### 3.8. Gastrointestinal Tract

**Figure 13:**
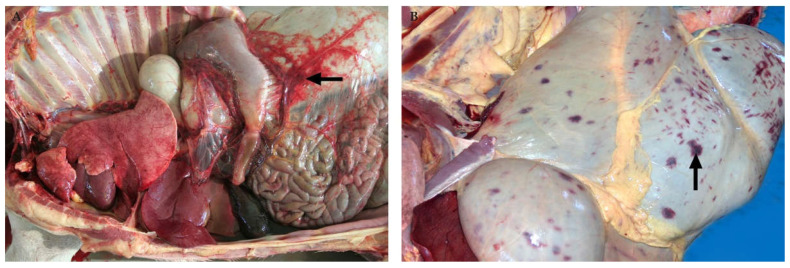
Omentum and rumen of an RVFV-infected adult sheep. (**A**) Marked congestion of the omental blood vessels with petechiae along the vessels and in the omental fat (arrow). (**B**) Petechiae and ecchymoses in the serosa of the rumen (arrow).

Occasionally in cattle and sheep, marked congestion of the mesenteric and omental vessels is present accompanied by petechiae and ecchymoses in the serosa along the entire course of the gastrointestinal tract (Figure 13) [35,45,82,86]. Haemorrhages may also be present in the mucosa and submucosa of the abomasum or the intestines [35,45,82]. Fresh or partially digested blood is also frequently present in the lumen of the abomasum or intestines in ruminants [35,82]. Histologically, small necrotic foci are occasionally present in the lamina propria of the small intestine [45,82]. In humans, intestinal haemorrhage, foci of necrosis in the mucosa and microscopic haemorrhages in the muscularis and subserosa have been reported [88].

In adult sheep, immunolabelling for RVFV is most often present in necrotic foci in the small intestine, either associated with cellular debris or on rare occasions in the cytoplasm of macrophages [45]. Occasionally, epithelial cells and vascular endothelial cells in the tongue label positively. In young lambs and sheep foetuses, viral antigen is prominent in endothelial cells in small blood vessels and capillaries, as well as vascular smooth muscle cells [37,46].

### 3.9. Subcutis and Skin

**Figure 14:**
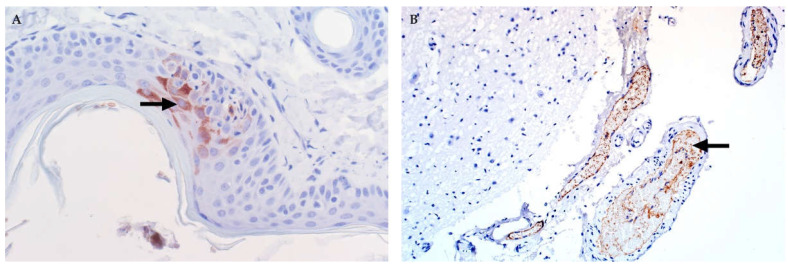
Skin and brain of RVFV-infected sheep (NovaRed IHC as detailed in Figure 5). (**A**) Immunolabelling in the epidermis (arrow) of an adult sheep, mag ×400. (**B**) Viral antigen in vascular endothelial cells and cellular debris in small blood vessels in the meninges (arrow) of a young lamb, mag 200×.

Subcutaneous haemorrhages are especially prominent on the abdomen, in the axillary region, the medial aspect of the hind limbs and the lower portions of the extremities [32,35,45,46,82,83,86]. Immunolabelling for RVFV in sheep is often present in keratinocytes or in superficial dermis in association with cellular debris or vascular endothelial cells (Figure 14A) [37,45,46].

### 3.10. Nervous System

Other than oedema, no lesions have been reported in any tissues from the central nervous system of natural cases in cattle or sheep [32,35,45,46,82,83,86]. Histologically oedema is especially prominent in foetuses [37]. In humans, focal areas of necrosis with an infiltrate of lymphocytes and macrophages may be present in the brain [88]. Perivascular cuffing indicative of encephalitis is also present in humans [88].

In sheep, viral antigen is mainly present in vascular endothelial cells or cellular debris in capillaries and small blood vessels in the meninges (Figure 14B). Occasionally, viral antigen is present in capillaries in the white or grey matter but not within the brain parenchymal cells. Non-cell-associated viral antigen is often present within the lumens of blood vessels in young lambs and sheep foetuses [45,46].

### 3.11. Reproductive Organs

Haemorrhages may be present in the testis of sheep [45]. While immunolabelling for RVFV is present multifocally within the connective tissue surrounding the seminiferous tubules, efferent ductules, and duct of the epididymis, in vascular smooth muscle, endothelial cells, macrophages, and fibroblasts, it is absent from the reproductive parenchyma [45]. Similarly, in the uterus of sheep haemorrhages are occasionally present in the perimetrium and myometrium yet viral antigen is confined to the blood vessels, and is always absent from the endometrium [45].

### 3.12. Placenta

**Figure 15:**
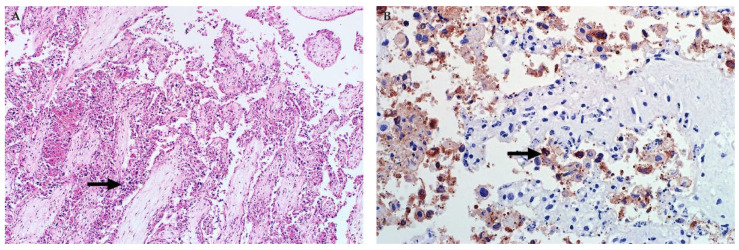
Placenta of RVFV-infected sheep. (**A**) Diffuse necrosis of trophoblasts with cellular debris (arrow) between the villi (H&E stain), mag 100×. (**B**) Immunolabelling in trophoblasts and cellular debris (arrow) in the cotyledonary chorioallantois (NovaRed IHC as detailed in Figure 5), mag 400×.

Placental lesions attributable to natural RVFV infection have only been described in sheep [37]. Macroscopic lesions include intercotyledonary oedema with congestion and necrosis of the cotyledons (Figure 15). Microscopically, oedema is prominent in the cotyledonary chorioallantois. The most significant histological lesion though is necrosis of trophoblasts and endothelial cells in the chorioallantois. In the cotyledonary villi, necrosis of trophoblasts is generally diffuse with multifocal cellular debris between the villi. Occasionally multifocal haemorrhages are present in the myometrium or the perimetrium or occur adjacent to chorioallantoic villi [37].

Immunolabelling for viral antigen is predominantly in trophoblasts and cellular debris in the cotyledonary chorioallantois (Figure 15) [37]. Occasionally binucleate and multinucleated maternal syncytial cells are also viral antigen positive. Viral antigen is also present in vascular endothelial cells, intravascular cellular debris, or non-cell associated. Labelling is also occasionally present in foetal blood vessels in the chorioallantoic membranes or the cotyledonary chorioallantois. Viral antigen is always absent from the squamous mesothelial cells lining the allantoic cavity and chorionic and allantoic mesenchyme [37].

## 4. Comparison of Contemporary Knowledge of RVF Disease with the Pathogenesis of Other Viral Haemorrhagic Fever Viruses

Rift Valley fever virus is one of many RNA viruses that causes viral haemorrhagic fever (VHF). The designation VHF is given to severe febrile illnesses that cause coagulation defects that can lead to bleeding and increased vascular permeability resulting in hypotension, shock, and death [98,99]. Many of these viruses are zoonotic, including RVFV, making them of particular concern from both a veterinary and public health perspective [1,100]. In human medicine haemorrhagic fever viruses are classified into four taxonomic families namely, the *Arenaviridae* (e.g., Lassa fever virus), *Bunyaviridae* (e.g., Crimean-Congo haemorrhagic fever virus and RVFV), *Filoviridae* (Ebola and Marburg viruses) and the *Flaviviridae* (dengue haemorrhagic fever virus and yellow fever virus) [101]. There appear to be many similarities between the pathology and pathogenesis of all the viral haemorrhagic fevers [99]. Therefore, some of what is unknown about the pathogenesis of RVF can be deduced from studies concerning other VHF viruses such as Ebola virus (EBOV), Crimean-Congo haemorrhagic fever (CCHF) virus (CCHFV), dengue haemorrhagic fever (DHF) virus (DHFV), Marburg haemorrhagic fever virus or Lassa fever virus. This is not a comprehensive review of the considerable data on all these agents but rather a comparison of a few of the better-studied diseases and their correlations to RVF.

### 4.1. Model of the Pathogenic Mechanism

**Figure 16:**
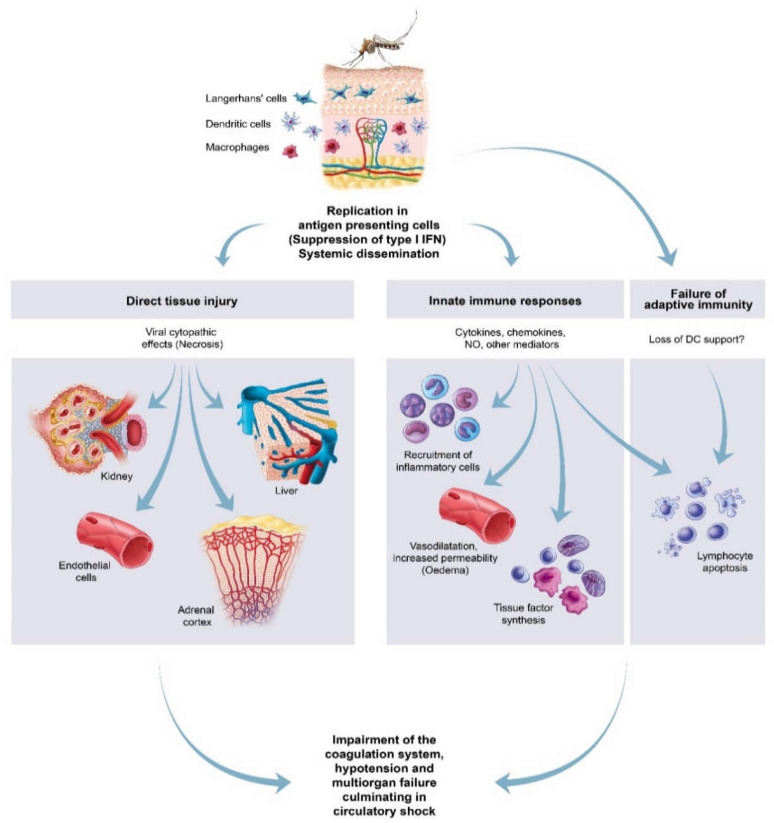
Model of the pathogenic mechanism underlying RVFV infection. Following a mosquito bite, the virus is endocytosed by antigen-presenting cells. Suppression of type I IFN production and necrosis of infected macrophages and dendritic cells cause wide dissemination of viruses. This systemic spread leads to necrosis in a variety of tissues and cells together with suppression of both the innate and adaptive immune responses. Apoptosis of lymphocytes might occur through mediator effects and loss of dendritic cell support, exacerbating the failure of the immune response. An excessive pro-inflammatory cytokine and chemokine response follow, resulting in increased microcirculatory dysfunction through the action of inflammatory mediators. Impairment of the coagulation system results in widespread haemorrhages. Fatal outcomes result from multiorgan failure, oedema in many organs (including the lungs and brain), hypotension and circulatory shock. DC, dendritic cell. NO, nitric oxide. (Illustration adapted from Bray M, 2005).

All VHFs are characterized by a broad spectrum of clinical manifestations ranging from asymptomatic cases to mild and severe symptomatic cases with malaise, fever, vascular permeability, decreased plasma volume, coagulation abnormalities and varying degrees of haemorrhage [100]. Common amongst the VHFs are liver damage, lymphocyte depletion, and abundant pro-inflammatory cytokine and chemokine production leading to systemic inflammatory response syndrome [99,100,102,103]. This may result in increased endothelial cell permeability with oedema, impairment of the coagulation system (as evidenced by thrombocytopenia, consumption of clotting factors, increased levels of fibrin degradation and bleeding), hypotension and multiorgan failure, culminating in circulatory shock in the terminal stages of disease (Figure 16) [100].

Detailed models representing the current understanding of VHF have been proposed prior [98,99,100]. Studies in nonhuman primates and rodents experimentally infected with EBOV, CCHFV, and DHFV, suggest that some of the antigen-presenting cells are early targets of these viruses [104,105]. Antigen-presenting cells are specialists at capturing microbial antigens, breaking them into small peptides, and displaying these to the appropriate T lymphocytes thereby inciting the adaptive immune response [106]. Antigen-presenting cells include dendritic cells of the dermis, spleen, and lymph nodes, Langerhans’ cells in the epidermis, macrophages, B lymphocytes, and type II and type III epithelioreticular cells of the thymus [106]. It is thought that early in the infection the virus is endocytosed by antigen-presenting cells in the mucosa or the skin [100,102]. Virus gains entry via the bite of an infected insect (e.g., RVFV, CCHFV, or DHFV), breaks in the skin (e.g., EBOV), or through exposure to excreta of infected rodents (e.g., Lassa fever virus) [1,100,104]. Virus replicates in the cytoplasm of macrophages or dendritic cells at the site of viral entry and is then conveyed to the lymph nodes and parenchymal cells in the liver, kidney, adrenal cortex, and other organs. Fragmentation of many cells via necrosis or apoptosis, with phagocytosis of cell remnants, promotes further systemic dissemination of virus [100,102]. Presumably, tissue macrophages and vascular endothelial cells become secondarily infected and systemic inflammatory response syndrome, accompanied by microcirculatory dysfunction and shock follows [102].

### 4.2. The Role of the Liver and Kidney

Hepatocellular death is common in all VHFs [102]. In RVF, the pathogenic mechanisms for hepatocyte death include apoptosis, induced by the presence of NSs as discussed earlier [78,107]. However, RVFV also induces the formation of nucleotide-binding domain, leucine-rich-containing family, pyrin domain-containing-3 (NLRP3) inflammasomes that activate caspase-1 leading to the maturation of interleukin 1β and interleukin-18 and the induction of pyroptosis [108]. The latter, in contrast to apoptosis, is a lytic cell death mechanism characterized by cell swelling, rupture of the plasma membrane, and cellular collapse [109,110].

In some cases of VHF, fulminant hepatic failure follows, however, hepatocellular lesions are often not significant enough to cause death [102]. Acute renal dysfunction may also play a role in the pathogenesis of VHFs with a fatal outcome. In the recent EBOV outbreak in Sierra Leone, patients had evidence of both hepatocellular damage and impaired kidney function and these were characterized by increased levels of liver enzymes, creatinine, blood urea nitrogen, and other markers [111]. Increased deviation from normal values for blood urea nitrogen (BUN), aspartate aminotransferase, and creatinine predicted a fatal outcome. In Marburg haemorrhagic fever, renal dysfunction presenting as proteinuria, with pale swollen kidneys, is frequently observed in fatal cases [112].

Similar findings are reported in RVF for both humans and ruminants. In a clinical study of severe illness due to RVFV infection in 165 human patients in Saudi Arabia, 69 were diagnosed with hepatic failure alone, 55 with both hepatic and renal failure, and 13 with renal failure alone, of which respectively 12, 39, and 3 patients died [41]. Acute renal failure associated with RVF was also described in hospitalized human patients in Sudan where 85 of 194 patients had signs and symptoms of renal failure without hepatic involvement [113]. Similarly, research comparing the susceptibility of three breeds of Nigerian sheep to experimental RVFV infection revealed increased BUN values in study participants starting on day three post-infection and continuing until the animals died [114]. Another experimental study also reported increased BUN values and histopathological changes indicative of renal injury in several sheep and cattle [94,97].

Renal dysfunction is also occasionally a serious complication of fulminant liver failure secondary to the development of portal hypertension, which then leads to splanchnic and systemic vasodilatation [115]. Vasodilatation, mediated by nitric oxide and other vasodilators, causes relative hypovolaemia and reduced effective central blood volume [115]. However vascular damage with plasma leakage causing non-dependant oedema and fluid sequestration in the body cavities may also exacerbate hypovolaemia and contribute to the development of renal failure. In DHF, the most severely affected children develop dengue shock syndrome due to excessive depletion of intravascular volume due to plasma leakage, accompanied by only minor bleeding manifestations, most commonly skin petechiae or bruising [116]. In Lassa fever, vascular damage with capillary leakage is also more prominent than haemorrhage, and results in pleural effusion, pericardial oedema, and hydropericardium in severe cases [117]. Similarly, in ovine neonates and foetuses that died from RVF, haemorrhages are minimal in most cases whereas brain and lung edema as well as body cavity effusions are common [37,46]. Hypovolaemia may also be further exacerbated by reduced synthesis of albumin by the liver and impaired secretion of steroid synthesizing enzymes by virus-infected adrenal cortical cells [102]. Adrenal cortical cells are permissive to infection by many VHF viruses including RVFV [37,45,46,94,95]. Reduced albumin leads to reduced plasma oncotic pressure and contributes to plasma leakage, whereas reduced levels of steroid synthesizing enzymes from the adrenal glands may contribute to hypotension and sodium loss [102]. Finally, renal impairment may also occur because of direct virus-related injury of kidneys, which has been demonstrated in both ovine and human RVFV infections [32,41,82,87,94,95,118].

### 4.3. Coagulation Disorders

VHFs also cause a diversity of coagulation disorders that present either as widespread bleeding or as thrombosis [99]. Humans and animals with severe illness due to RVFV may have bleeding from the gums, haematemesis, haemoptysis, epistaxis, melaena, haematuria, vaginal bleeding, petechial rashes and ecchymoses of the skin, or bleeding from venipuncture sites [29,41,45,86,119,120]. Conversely, thrombosis is described in earlier studies in ruminants, wherein mention is made of thrombosis of the central veins of the liver in 3 of 34 lambs, and fibrin thrombi in 6 of 30 adult cattle and calves [35,82]. Coronary thrombosis was also reported in one human patient in 1951, but cardiovascular disease was not excluded in this patient who also had a ‘tight feeling over the chest’ and refused hospitalization [121]. In contrast, lesions that were described in four patients that died from RVF during the outbreak in 1975 in South Africa did not mention microthrombi in any of the tissues [88]. Additionally, there was no convincing evidence of vascular fibrinoid necrosis or microvascular fibrin thrombi in any organ in recent studies of natural sheep cases [37,45,46]. Therefore, evidence of thrombosis in RVF is contradictory.

The mechanisms of widespread bleeding in VHFs have often been attributed to direct viral infection or damage of vascular endothelial cells and thrombocytopenia [99]. Thrombosis has been ascribed to the release of pro-inflammatory cytokines and chemokines from virus-infected endothelial cells and monocytes/macrophages [99]. Disseminated intravascular coagulation (DIC) possibly follows, because of the activation of the coagulation cascade and a reduction in the production of coagulation factors due to severe hepatic necrosis. However, DIC can be classified as either enhanced-fibrinolytic-type DIC (fibrinolytic DIC) or suppressed-fibrinolytic-type DIC (thrombotic DIC) [122].

In thrombotic DIC (e.g., in sepsis) multiple microthrombi form in circulation due to coagulation cascade activation [122]. However, because plasminogen activator inhibitor is overexpressed in the vascular endothelium, fibrinolysis is suppressed and many microthrombi remain, causing multi-organ failure [122]. Laboratory findings include an elevation in thrombin–antithrombin complex (TAT), a coagulation activation marker, and only mildly elevated plasmin-α2 plasmin inhibitor complex (PIC), a fibrinolysis activation marker. Fibrin/fibrinogen degradation products (FDPs) and D-dimer levels are also elevated, and bleeding complications are relatively mild in thrombotic DIC. In contrast, in fibrinolytic DIC, microthrombi are histologically difficult to demonstrate due to enhanced fibrinolytic activation [122]. Laboratory findings include a steep increase in PIC, but only a mild increase in the activity of plasminogen activator inhibitor, the fibrinolytic inhibitory factor [122]. Additionally, levels of TAT, D-dimers and FDPs, which reflect the dissolution of microthrombi, are also elevated. Bleeding symptoms in enhanced fibrinolytic DIC are severe, and life-threatening bleeding may occur [122].

The occurrence of DIC in human RVF cases in Saudi Arabia has been reported but was inadequately characterized to enable classification as either fibrinolytic or thrombotic [5,41]. More recently though, a study of 3 human RVF cases in Uganda demonstrated elevated D-dimer and tissue plasminogen activator levels, which is consistent with increased fibrinolysis [123]. In DHF, it was demonstrated that levels of FDPs are not elevated to a degree consistent with thrombotic DIC [116]. Instead, fibrinolytic DIC occurs in DHF wherein degradation of fibrinogen prompts secondary activation of procoagulant homeostatic mechanisms [124]. In sheep, experimentally infected with RVFV, thrombocytopenia and prolonged prothrombin and clotting times are present and plasma fibrinogen levels fluctuate during the period of RVFV infection [114]. In a wild-type RVFV rhesus macaque infection study, 3 of 15 animals had haemorrhagic disease accompanied by thrombocytopenia, prolonged prothrombin and clotting times and significant decreases in FDPs and fibrinogen levels, again consistent with fibrinolytic DIC [125]. Consequently, it seems unlikely that microthrombi play a significant role in the pathogenesis of RVF.

### 4.4. Suppression of the Immune System

Dysregulation of the inflammatory response also contributes to a fatal outcome in VHF. Wild-type RVFV infection of human monocyte-derived macrophages can lead to a productive infection and inhibition of the innate immune response via decreased expression of IFN-α2, IFN-β, and TNF-α [126]. In human patients infected with RVFV, interleukin-8 (IL-8) and interleukin-6 (IL-6) levels were increased and were at similar levels in fatal cases and survivors [123,127]. Interleukin-8 is a pro-inflammatory chemokine produced by macrophages in response to infection, while IL-6 is an important mediator of fever [127]. Serum levels of monokine-induced-by-gamma-interferon (MIG), interferon-gamma-induced-protein-10 (IP-10), and interleukin-10 (IL-10) were significantly increased while the chemokine RANTES (regulated-upon-activation-normal-T-cell-express-sequence) was significantly decreased in fatal human RVFV cases relative to survivors and controls [127]. This suggests an imbalance in the immune response in fatal RVFV cases, because MIG, IP-10, and RANTES are pro-inflammatory chemokines whereas IL-10 is immunosuppressive. This imbalance would contribute to the failure of adaptive immune responses to clear the infection in fatal cases and explain why RVFV replicates to significantly higher levels in patients with fatal outcomes, but the details of such a model remain to be determined [127,128].

### 4.5. Lymphocytes

Interestingly none of the VHF viruses infects lymphocytes. However, their rapid loss by apoptosis is a prominent feature of these diseases and lymphocytolysis is often noted [100,103,129]. In Ebola subtype Zaire, MARV and RVFV infections, lymphoid depletion affects the centres of B-cell follicles in lymph nodes [45,129]. Fatal outcomes in humans infected with Ebola subtype Zaire are also characterized by very low levels of circulating cytokines produced by T lymphocytes and by the massive loss of peripheral CD4+ and CD8+ lymphocytes, causing a profound suppression of the adaptive immune responses [103]. However, the innate immune responses are also impaired by Ebola subtype Zaire infection which is characterized by hypersecretion of numerous pro-inflammatory cytokines, chemokines, and growth factors, and by the significant absence of a type I interferon production [103]. The cause of lymphocytolysis in VHF’s is unknown and further study of the pro-inflammatory cytokine milieu and its potential relationship to the observed lymphocytolysis caused by natural RVFV infections is warranted, because laboratory animal models such as the mouse have produced contrary findings [127].

### 4.6. The Central Nervous System

Encephalitis, a complication of RVF in humans, has been experimentally reproduced in mice and rats, but has not been described in sheep or any other ruminants [41,130,131]. Neurologic disease has also been described in other VHFs such as Lassa fever, DHF, and Marburg haemorrhagic fever [112,132,133]. Meningoencephalitis associated with RVFV infection was described in 7 of 165 patients in Saudi Arabia and accompanied by either retinitis (*n* = 5), hepatitis (*n* = 3), or kidney failure (*n* = 1) [41]. In experimental infection studies in marmosets and African green monkeys, RVFV also causes encephalitis [134]. Previously, in fatal human RVF cases, focal necrosis with an infiltrate of mononuclear cells and perivascular cuffing in the central nervous system (CNS) has been described [88]. This finding together with the delayed-onset encephalitis and/or retinitis in humans suggests an inadequate adaptive immune response [41,135]. Additionally, an increased mortality rate is seen in human patients infected with both RVFV and human immunodeficiency virus (HIV), and all these patients present with CNS symptoms [136]. Trafficking of the virus across the blood-brain barrier, a layer of vascular endothelial cells reinforced by tight junctions between the cells and supported by astrocytes and pericytes, might be blocked by RVFV-neutralizing antibodies [131]. Experiments in wild-type mice and monocyte recruitment deficient CCR2 knockout mice, variously depleted of CD4+ and CD8+ T lymphocytes and infected with NSs gene deleted RVFV, demonstrated that both CD4+ and CD8+ T cell populations and monocytes were critical for prevention of RVFV-neurologic disease and that this immune control occurs in the periphery not the central nervous system [135]. Furthermore, as shown by markedly decreased neutralizing antibody responses and increased viral loads in the CD4+ lymphocyte depleted mice, activation of CD4+ T lymphocytes is requisite to augment RVFV antibody affinity maturation and class switching in B lymphocytes [137]. Therefore, individuals with CD4+ T lymphocyte dysfunction might be at an increased risk of developing severe RVF, including encephalitis.

Notably, it has not been established exactly how RVFV crosses the blood-brain barrier. Aerosol or intranasal exposure might be necessary since encephalitis occurs in laboratory animals exposed to RVFV via this route rather than via subcutaneous injection [107,138,139]. Furthermore, although RVFV is mosquito-transmitted, consuming or handling products from sick animals, including slaughtering sick livestock, and handling dead foetuses is associated with severe disease or death [4,5,6]. Therefore, RVFV might infect peripheral sensory and motor neurons in the eyes or oronasal mucosa directly, causing encephalitis and retinitis via these routes [140]. Immunocompetent Lewis rats exposed to wild-type RVFV by aerosolization develop lethal encephalitis and have low levels of viral RNA in their eyes [141]. Lethal disease in these rats is also accompanied by increases in monocyte chemoattractant protein-1 (MCP-1), macrophage colony-stimulating factor (M-CSF), keratinocyte chemoattractant/human growth-regulated oncogene (Gro/KC), RANTES, and IL-1β, all evident in the serum before the onset of clinical disease, and these increases are followed by an inflammatory derangement in the brain during clinical illness [141]. It has been hypothesised that these chemokines may be actively recruiting monocytes, macrophages, and neutrophils into the CNS [141]. Additionally, aerosol exposure of rats to wild-type RVFV has demonstrated that neurological symptoms commence concomitant with increased vascular permeability and possible brain oedema, and that virus replication occurs in the brain several days before vascular leakage is detected [142]. Therefore, in some species at least, RVFV seemingly enters the CNS via olfactory neurons where replication of virus causes inflammation and increased vascular permeability, with concomitant release of chemokines that attract mononuclear cells to the CNS and cause neurological symptoms. Unfortunately, though, this still does not explain why ruminants have never been reported to develop encephalitis, since it seems likely that they would encounter aborted foetuses and placentas.

## 5. Conclusions

Outbreaks of RVF occur virtually every year in Africa in one or more countries. However, the disease is often diagnosed in humans before cases in livestock are noted. Ideally, animals should serve as sentinels to minimize loss of human life. To successfully diagnose RVF in ruminants that died from the disease, ideally tissues from multiple age groups should be examined. Abortions in livestock are often the first indication that RVFV is in circulation. However, diagnosing RVF in foetuses is challenging when samples from the ewe or the placenta are not available, because foetal organ samples are often negative for virus. A diagnosis may also be challenging during an interepidemic period when a limited number of deaths and abortions occur, and the disease is not expected to be present. Consequently, continued virus surveillance in endemic areas, where RVFV infection is a plausible differential diagnosis in the interepidemic periods, is warranted. Appropriate samples should be taken, and suitable additional tests requested.

Much is still unknown about the pathogenesis of RVF, warranting further research in ruminants and animal models of human disease. Various components in the clotting cascade should be studied to determine whether fibrinolysis and the formation of fibrin thrombi occur concomitantly in RVF, thereby preventing overt thrombosis and infarction in most cases. The possibility that SCARA1 is a cellular receptor for RVFV that might play a role in successive rounds of RVFV infection in various tissues, also warrants further investigation. It is also important to investigate RVFV induced changes in the levels of various lymphocyte subsets and the circulating cytokines produced by them. Specifically, it should be determined which subset of lymphocytes is worst affected, at what time-point post-infection this occurs, and how this affects the adaptive immune response. The finding that RVFV infects cells in the testis raises the question of whether the virus can be sexually transmitted. Studies in ruminant models might show if viable virus is present in semen, and if confirmed, how long individuals potentially remain infectious. Lastly a clear understanding of why RVFV induced encephalitis occurs in humans but not in ruminants might provide a course of action to prevent this devastating complication of RVF.

## Data Availability

Data is contained within the article.
